# Observations of Drinking Water Access in School Food Service Areas Before Implementation of Federal and State School Water Policy, California, 2011

**DOI:** 10.5888/pcd9.110315

**Published:** 2012-07-05

**Authors:** Anisha I. Patel, Kumar Chandran, Karla E. Hampton, Kenneth Hecht, Jacob M. Grumbach, Amanda T. Kimura, Ellen Braff-Guajardo, Claire D. Brindis

**Affiliations:** Author Affiliations: Kumar Chandran, Kenneth Hecht, Ellen Braff-Guajardo, California Food Policy Advocates, Oakland, California; Karla Hampton, ChangeLab Solutions formerly known as Public Health Law & Policy, Oakland, California; Jacob Grumbach, Amanda Kimura, Philip R. Lee Institute for Health Policy Studies, University of California, San Francisco, San Francisco, California; Claire D. Brindis, Department of Pediatrics and the Philip R. Lee Institute for Health Policy Studies, University of California, San Francisco, San Francisco, California.

## Abstract

**Introduction:**

Recent legislation requires schools to provide free drinking water in food service areas (FSAs). Our objective was to describe access to water at baseline and student water intake in school FSAs and to examine barriers to and strategies for implementation of drinking water requirements.

**Methods:**

We randomly sampled 24 California Bay Area public schools. We interviewed 1 administrator per school to assess knowledge of water legislation and barriers to and ideas for policy implementation. We observed water access and students’ intake of free water in school FSAs. Wellness policies were examined for language about water in FSAs.

**Results:**

Fourteen of 24 schools offered free water in FSAs; 10 offered water via fountains, and 4 provided water through a nonfountain source. Four percent of students drank free water at lunch; intake at elementary schools (11%) was higher than at middle or junior high schools (6%) and high schools (1%). In secondary schools when water was provided by a nonfountain source, the percentage of students who drank free water doubled. Barriers to implementation of water requirements included lack of knowledge of legislation, cost, and other pressing academic concerns. No wellness policies included language about water in FSAs.

**Conclusion:**

Approximately half of schools offered free water in FSAs before implementation of drinking water requirements, and most met requirements through a fountain. Only 1 in 25 students drank free water in FSAs. Although schools can meet regulations through installation of fountains, more appealing water delivery systems may be necessary to increase students’ water intake at mealtimes.

## Introduction

Growing research implicates sugar-sweetened beverages (SSBs) such as sodas and sports drinks as a contributor to rising obesity rates (1-3). Nationally, 80% of children aged 2 to 19 years consume at least 1 SSB daily (4). Policy makers have responded with legislation to restrict sales of unhealthy beverages in schools (5). However, less emphasis has focused on improving consumption of healthy alternatives, namely water.

Substituting water for SSBs can result in an average decrease of 235 calories per day (6) and can also prevent obesity (7-10). In a randomized controlled trial of German elementary schools, installation of filtered, refrigerated fountains, distribution of reusable water bottles, and promotion of water intake through education decreased the risk of overweight among intervention study participants (9).

In September 2010, California enacted SB 1413, legislation requiring schools to provide access to free drinking water during mealtimes in school food service areas (FSAs), locations where meals are served or eaten, by July 1, 2011 (11). In December 2010, the President signed the Healthy, Hunger-Free Kids Act of 2010 (Act), which included a similar provision requiring access to free drinking water in US public schools where meals are served (12).

To our knowledge, no data have been published regarding water access in school FSAs, and such information would be useful to schools seeking to implement new water requirements. The objective of this study was to use school observations to document provision of water and student water consumption patterns in FSAs in a sample of California Bay Area schools. Secondary objectives were to document barriers to water provision in schools and to identify innovative water consumption promotion practices in schools.

## Methods

### Study participants

We collected data from January to May 2011 before implementation of SB 1413 and the Act. We selected public schools from the National Center for Education Statistics’ Common Core of Data (CCD) (13). The 1,313 standard California public schools (excluding private, K-8 or K-12, special educational, vocational, and alternative schools) were from California’s Bay Area region (Alameda, Contra Costa, Marin, Napa, San Francisco, San Mateo, Santa Clara, Solano, and Sonoma counties). To understand water provision in schools of varying type and location, we stratified eligible schools by CCD urban-centric locale and school type. We collapsed locales (large, midsize, or small cities; large, midsize, or small suburbs; fringe, distant, or remote towns; and fringe, distant, or remote rural areas) into 4 categories: city, suburb, town, and rural area. In these categories, we further stratified schools by primary, middle, and high school to generate 12 separate sampling strata (eg, city high, city middle, rural elementary). Using a random number generator, we generated a random list of all eligible California schools for each of the 12 strata.

### Instruments

We developed a telephone survey and observational tool to assess provision of drinking water and policies and practices related to school drinking water on the basis of a literature review, a previous qualitative study conducted by the first author (A.I.P.) on access to school drinking water (14), and feedback from study collaborators. To improve the content validity of the survey and observational tool, we used a snowball sampling approach to identify 15 experts with content knowledge of drinking water access and policies and practices related to drinking water access in schools. We used these experts’ comments to revise the survey and observational tool, which we then pilot tested with 10 staff from ineligible schools (ie, located outside of the Bay Area or nonstandard public schools).

The observational tool assessed the following domains: free drinking water access and quality, student drinking water intake in FSAs, and bottled water and other beverages available for purchase. We also reviewed school documents to assess school drinking water policies.

Two observers used the tool to code access to free drinking water, characterized as the school locations where drinking water was available (ie, FSAs) and the type of water source available (eg, fountains, water dispensers). Observers also documented drinking water quality by coding the temperature (1 = very cold, 2 = cold, 3 = room temperature, 4 = warm), clarity (1 = clear, 2 = cloudy, 3 = yellow, 4 = brown), and flow strength (1 = high, 2 = medium, 3 = low, 4 = none) of free drinking water in school FSAs. Observers also characterized the cleanliness of the drinking water sources (1 = very clean, 2 = clean, 3 = unclean, 4 = very unclean).

Observers assessed student intake of water in FSAs by counting the number of students who drank free drinking water in the FSA during all lunch periods on an observation day. Although students may eat lunch in locations throughout the campus, it is impractical to ensure that drinking water is accessible in all of these locations. For these reasons, we defined an FSA as an area within 100 feet of where reimbursable meals were served. The water source observed was the one closest to the FSA. We estimated the percentage of students observed drinking water at lunch by dividing the number of students observed drinking water at lunch by the daily attendance.

Observers also recorded the type, location, and cost of beverages provided with the school lunch and available for purchase. After the visit, we obtained wellness policies from each participating school’s district for analyses of language regarding drinking water provision.

The school administrator survey measured drinking water availability (ie, type, location, and source), drinking water policies and practices (eg, quality and fountain accessibility or maintenance), and barriers (eg, cost, policies and contracts, student behavior) to school drinking water access. We obtained school sociodemographic data from the Education Data Partnership (15).

### Data collection procedures

We mailed an invitation letter to principals of eligible schools. A research assistant (RA) then contacted study participants to confirm receipt of materials, answer questions, and schedule an interview time. RAs contacted potential study participants until they declined. If a school declined, we sampled the next randomly chosen school from the study stratum. To obtain our goal sample size of 24 schools, we had to contact 44 schools from the overall sampling frame of randomly selected California schools. We obtained consent from survey respondents (eg, school principals, school administrators) before collecting data.

RAs or the first author (A.I.P.) conducted all surveys. We audio recorded surveys, which lasted 10 to 20 minutes. After conducting the surveys, RAs scheduled an observational visit. Each RA had extensive training using the observational protocol before data collection; 2 RAs and the principal investigator (A.I.P.) visited schools until consistency in coding was achieved (κ > 0.80) for all variables. Thereafter, 2 RAs visited each school and simultaneously coded all observational data on paper checklists. Kappa statistics ranged from 0.88 to 1.0 for observational variables, indicating excellent interobserver reliability.

We provided study participants with $50 gift cards for participation. University of California, San Francisco’s Committee on Human Research approved the study.

### Data analysis

We analyzed data using Stata version 11 (StataCorp LP, College Station, Texas). We used descriptive statistics (frequencies, percentages, and means) to summarize school characteristics (ie, school population, racial/ethnic breakdown, percentage of students eligible for free and reduced-price meals through the National School Lunch Program [NSLP]), and main study outcomes (eg, drinking water availability in FSAs and students’ water intake). We used *t* tests and χ^2^ tests to assess the differences between study participants and nonparticipants.

## Results

Our response rate was 55%. Of schools that declined (n = 20), 13 declined due to lack of time, 5 stated no reason for declining, and 2 declined due to lack of interest. Participating schools did not differ significantly from schools that declined in terms of school type, urban-centric locale, mean student enrollment, percentage of students who qualified for free or reduced-price meals, student race/ethnicity, or percentage of English learners (Table 1). Sociodemographic characteristics of study schools were similar to those of Bay Area schools in aggregate. Of the 24 administrators who participated, 16 were principals, 3 were assistant principals, and 5 were “other.”

**Table 1:** Characteristics of Observation Schools Compared With Bay Area and California Schools^a,b^

Characteristic	California Schools (n = 9,888)	Bay Area Schools (n = 1,747)	Observation Schools (n = 24)	Schools That Declined Participation (n = 20)
**School type, n (%)**				
Elementary	5,736 (58)	1,030 (59)	8 (33)	6 (30)
Middle	1,305 (13)	245 (14)	8 (33)	7 (35)
High	1,264 (13)	227 (13)	8 (33)	7 (35)
**School locale, n (%)**				
Rural	1,582 (16)	70 (4)	6 (25)	4 (20)
Town	890 (9)	35 (2)	6 (25)	2 (10)
Suburb	3,460 (35)	646 (37)	6 (25)	11 (55)
City	3,856 (39)	996 (57)	6 (25)	3 (15)
**Student enrollment, mean no.**				
Elementary	530	475	385	519
Middle	810	741	687	682
High	1,402	1,247	1,644	1,481
**Eligible for free or reduced-price meals, n (%)**	3,404,790 (55)	382,353 (39)	8,254 (38)	6,936 (38)
**API^c^ growth score**	767	NA	801	804
**Race/ethnicity, n (%)**				
African American	424,327 (7)	78,431 (8)	1,521 (7)	1,095 (6)
Latino	3,118,404 (50)	323,530 (33)	7,603 (35)	6,206 (34)
White	1,673,278 (27)	284,314 (29)	6,951 (32)	7,119 (39)
Asian/Pacific Islander	720,311 (12)	225,490 (23)	3,910 (18)	3,468 (19)
**English learners, n (%)^d^**	1,468,771 (24)	215,686 (22)	4,127 (19)	3,651 (20)

Abbreviation: NA, not applicable.

^a^ Participating schools did not differ statistically from schools that declined in terms of school type, urban-centric locale, mean student enrollment, percentage of students who qualified for free/reduced price meals, student race/ethnicity, or percentage of English learners (*P* > .05).

^b^ Data obtained from Education Data Partnership (15).

^c^ API refers to the Academic Performance Index, a measure of academic performance in California schools based on standardized testing. The score ranges from 200 to 1,000, with a target score of 800 for California.

^d^ Students who report a primary language other than English and who have been determined by the state of California to lack clearly defined English language skills necessary to succeed in the school’s regular instructional programs.

### Beverages available in food service areas

Of the 14 schools that offered free drinking water in FSAs, 10 offered water via fountains, while the remaining schools had an alternative delivery system (Table 2). Beverages most commonly offered with the NSLP included 1%/skim unflavored and flavored milk; 100% juice and bottled water were offered in some schools. Only 1 elementary school offered competitive beverages or beverages for purchase in the FSA. In secondary schools, the most commonly available competitive beverages were bottled water and sports drinks. The mean price of bottled water in FSAs was $0.92 per bottle. Despite legislation (Senate Bill 965) that since 2009 has prohibited the sale of SSBs other than sports drinks in California public schools, 1 school offered “slushies,” a frozen flavored beverage.

**Table 2:** Beverages Available in Observation Food Service Areas,^a^ by School Type (n = 24)

Beverage type	Elementary (n = 8)	Middle (n = 8)	High (n = 8)
**Free water**			
Fountains	5	3	2
Other^b^	0	2	2
**Available via the National School Lunch Program**			
Bottled water	1	1	0
1%/Skim unflavored milk	8	8	8
2%/Whole unflavored milk	0	0	0
Flavored milk	5	6	7
100% Fruit juice	2	1	0
**Competitive and for purchase**			
Bottled water	0	7	7
1%/Skim unflavored milk	0	0	0
2%/Whole unflavored milk	0	0	0
Flavored milk	0	0	1
100% Fruit juice	1	7	6
Sports drinks	0	6	5
Other sugar-sweetened beverages	0	0	1
Noncaloric drinks	0	0	1

^a^ Food service area refers to the area in which meals are served and/or eaten. When schools allowed students to eat anywhere on campus we defined the food service area as within 100 feet of the location where food was served.

^b^ Other refers to sources of free water other than drinking fountains (eg, water dispensers, bottled water, hydration stations, pitchers).

### Student water intake in food service areas

**Figure 1:**
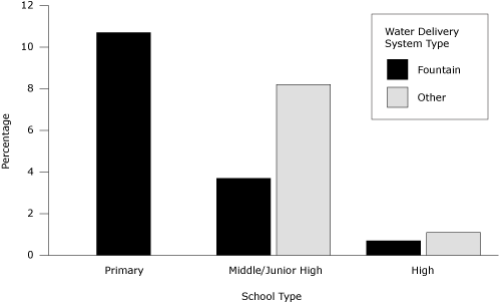
Percentage of students (n = 11,226) observed drinking free water at lunch, by school type and water delivery system, Bay Area, California, 2011. The percentage of students drinking water at lunch was obtained by counting the number of students who drank water in the food service area divided by the total daily student attendance. There were no nonfountain sources of drinking water in primary schools. The percentage of middle school students who drank water from a fountain source was significantly lower than the percentage of students who drank water from a nonfountain source (*P* = .04). This difference was not significant among high school students (*P* = .09). The percentage of students drinking water at lunch was higher when water was available via a delivery system other than a drinking fountain (eg, water dispenser with cups).

At schools with free water in FSAs, only 4% of the 11,226 students in daily attendance were observed drinking free water at lunch. The percentage was highest in elementary schools, followed by middle and then high schools (Figure 1). In the schools that did provide water in FSAs, most provided water of good overall quality; the mean water temperature was cold (1.9), the mean water clarity was clear (1), mean water flow was of medium strength (1.8), and mean cleanliness of the water delivery system was very clean to clean (1.6). Only 1 school dispensed water that was not cold or very cold and only 2 schools dispensed water of low or no strength. Although all water delivery systems in school FSAs were described as clean, 2 fountains contained gum and 1 fountain contained a small amount of dirt.

**Table:** 

School Type	No. (%) of Students Accessing Water Delivery System	
	Fountain	Other^a^
**Primary**	246 (10.7)	NA
**Middle/junior high**	72 (3.7)	116 (8.2)
**High**	24 (0.7)	22 (1.1)

Abbreviation: NA, not applicable.

^a^ Includes hydration stations, dispensers, and coolers.

### Alternative drinking water sources in food service areas

**Figure 2:**
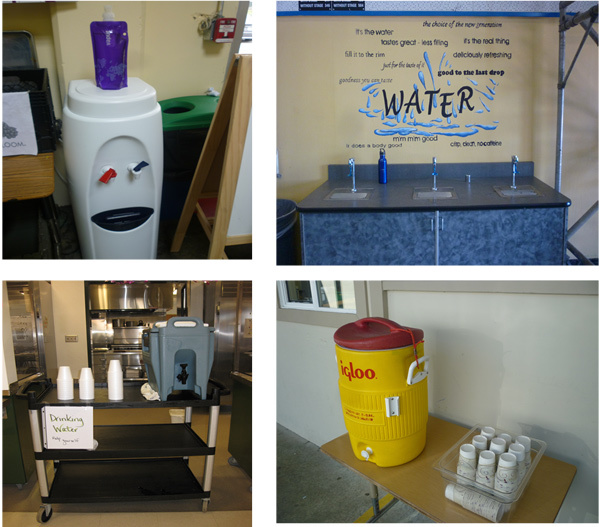
Alternative drinking water sources in Bay Area, California, food service areas. Top left, a hot and cold water dispenser found within 10 feet of where food was served in an indoor high school cafeteria. Resting on the dispenser is a purple Vapur-brand foldable reusable water bottle sold as a fundraiser by a student group at the school. Top right, a hydration station located approximately 50 feet of where food was served in an indoor high school cafeteria. Bottom left, a Cambro-brand cooler and foam cups located within 5 feet of where food was served in an indoor middle school cafeteria. Bottom right, an Igloo-brand cooler and Dixie-brand cups provided for students outdoors within 5 feet of a food service window but approximately 100 feet from the main cafeteria in a middle school.

We photographed alternative water delivery systems in school FSAs (Figure 2). Only 4 schools, all secondary, offered water through a nonfountain source. One high school purchased a dispenser that dispensed filtered hot and cold tap water. According to the school, the dispenser was installed because no other functional drinking water source was available in the school. A school administrator estimated that the unit, paid for by the nonprofit organization that operates the charter school, costs $200 to $400. Filter changes required every few months were estimated as $20 per change. Students were expected to bring their own container to access water from the dispenser. At this same school, 1 student was selling a plastic foldable bisphenol A (BPA)–free water bottle at the school as a fundraiser ($4 per bottle).

Another high school installed a hydration station with a mural backsplash displaying promotional messages to encourage water intake. The hydration station was installed by the food service director, a champion of health and nutrition. The cost of the station, estimated at $2,000, was paid for through the food services budget, which often covers ancillary costs (eg, trays, napkins) associated with meal service. Students at this school, similar to the school with a dispenser, were expected to bring their own containers for drinking water due to concerns about waste (eg, cups) not being discarded appropriately in trash receptacles.

### School administrator perceptions regarding new school drinking water requirements

Half (12 of 24) of school administrators had heard about the Act or SB 1413. In our study, 14 schools had water in the FSA before implementation of SB 1413 and the Act. Of these 14 schools, 3 reported that they began providing water in the FSA as a result of hearing about SB 1413. Administrators most commonly cited more pressing academic concerns (n = 19) and the cost of new water programs (n = 16) as barriers to implementation of water requirements.

As a result of hearing about SB 1413, food services departments in 2 middle schools filled water dispensers with ice water and provided water with disposable cups at lunch. At both of these schools, dispensers were on hand at the school, and food services absorbed the cost of cups. Per estimates, water coolers cost approximately $30 each and cups are $0.02 to $0.05 each for 4- to 6-ounce foam cups and Dixie-brand cups.

### Water language in school district wellness policies

Among wellness policies for the 20 school districts represented in our study (4 schools were in duplicate districts), 11 included language related to drinking water (Table 3), but none specifically mentioned that free drinking water should be available in FSAs. Three district wellness policies contained water-related language in more than 1 thematic area (eg, water allowable as a competitive beverage, provision of water with snacks). Only half of schools that offered water via a nonfountain source had water-related language in their wellness policy.

**Table 3:** Language Regarding Drinking Water Provision in Public School District Wellness Policies, Bay Area, California

Water-Related Theme	No. of Districts With Theme	Example from Wellness Policy
Water without added sweeteners is allowable in schools as a competitive beverage	10	From one-half hour before to one-half hour after the end of the school day, the only beverages sold to pupils by any entity are: fruit- or vegetable-based drinks of no less than 50% fruit or vegetable juice and no added sweeteners; water with no added sweeteners; milk (2%, 1%, nonfat, soy or rice, and other nondairy milk); or electrolyte-replacement beverages containing no more than 42 grams of sugar per 20-ounce serving.
		Any student organization or organizations may be approved to sell food at any time during the school day, including the regularly scheduled food service period(s) as provided in 1) and/or 2) below: only 1 such organization each school day selling no more than 3 types of food or beverage items such as healthy snacks, popcorn, nuts, fruit, fruit juices, and water.

Marketing and promotion of healthful foods and beverages such as water during the school day and at school-sponsored events and activities	3	Marketing activities that promote healthful behaviors (and are therefore allowable) include vending machine covers promoting water; pricing structures that promote healthy options in á la carte lines or vending machines.
		Healthy food and beverage choices (ie, fresh fruits and vegetables, whole grains, low-fat dairy products, 100% fruit juice, and water) will be promoted in all school activities and school-sponsored events where food and beverages are offered or sold.

Emphasis on serving water with snacks at school	2	Snacks served during the school day or in after-school care or enrichment programs will make a positive contribution to children’s diets and health, with an emphasis on serving fruits and vegetables as the primary snacks and water as the primary beverage.

Request that donated drinks for parties and school events include water	1	Schools will request that donated drinks (under any existing soda contract, and brought in for class parties, school sponsored events, etc) will be from the list below: water, 100% fruit juice or fruit-based drinks with no less than 50% fruit juice and no added sweetener, electrolyte-replacement beverages with no more than 42 grams of added sweetener per 20-ounce serving, and/or nonfat or reduced fat milk.

## Discussion

This study, conducted after enactment but before implementation of federal and state requirements regarding water in FSAs and the first peer-reviewed study to examine water access in school FSAs (16), demonstrated that nearly half of schools did not have free water available in FSAs and that drinking fountains were the most common water source.

Observations of students indicated that the percentage of students drinking water in FSAs was higher in schools with younger children and among schools with nonfountain sources of water. As suggested in previous studies, students may choose not to drink from fountains because they perceive water from fountains as unclean or unsafe to drink or because fountains have genuine problems (eg, unclean, in disrepair, dispense unpalatable water, permit only small sips) (14,17). Because fountains in this study were in good condition, we hypothesize that low student intake of water from fountains may be due to student preferences for other beverages or for water from alternative drinking water delivery systems, rather than because of poor water quality. Previous studies suggest that providing appealing water may increase student water intake (9,18). Further understanding what types of water delivery systems are most appealing to students of different ages is essential to increasing students’ water intake.

Tap water from a fountain was the most common source of free water available in study school FSAs. Only a few schools offered free bottled water with meals. In most secondary schools, bottled water was available as a competitive beverage (19,20). The price of bottled water ($0.92 per bottle) could prevent students, particularly those of lower socioeconomic status, from purchasing water at school on a frequent enough basis to meet recommendations for adequate water intake.

Given that a large number of schools did not have free water in FSAs before the corresponding legislation went into effect, schools may need assistance in meeting the requirements. A major barrier is a lack of knowledge of drinking water requirements among school administrators. Although SB 1413 and the Act passed in fall of 2010 and received media coverage, many administrators were not aware of the legislation. One contributing factor could be that federal and state and agencies (US Department of Agriculture, California Department of Education) did not provide schools with guidance until April 2011 (21,22). However, partnering with statewide associations of teachers, school nurses, as well as school boards may be an effective strategy for disseminating legislative information.

Because schools participating in federal child nutrition programs (eg, NSLP) must implement school wellness policies, which include goals and action steps for school-based activities that promote student wellness, such policies can be leveraged to assist schools in improving FSA drinking water provision. In our analysis, no wellness policies mentioned provision of free drinking water in school FSAs and only 3 policies included language about drinking water provision that encompassed more than 1 theme. No studies have examined wellness policies for language regarding school FSA water access. A 2008–2009 national study of school wellness policies showed that only 12% to 13% of schools had language regarding free water availability throughout the school day (23). On the basis of these limited studies, developing more comprehensive school wellness policy language regarding water access may help improve drinking water access and intake in schools (24).

However, having comprehensive water language in school wellness policies may not be sufficient to ensure that safe and appealing drinking water is available on school campuses. Ongoing implementation and monitoring is needed to ensure continued access to free drinking water. As seen in this study, schools that provided a nonfountain water source often had a “water champion” who was essential to developing and sustaining drinking water programs. These champions, who were all food service directors in this study, went beyond the letter of the law to provide students with water that was more appealing than water provided via a fountain. Prioritizing drinking water for school-level policy bodies such as wellness committees or coordinated school health councils may help to institutionalize such water champions. Because water is a topic that spans multiple disciplines, parents, facilities managers, teachers, and food services directors can all champion water in schools, preferably working together toward achieving this shared aim.

This study has several limitations. Although the use of observations instead of self-report is a strength, the observational methods limited the sample size and confined the study to a single California region. Another limitation is the poor response rate. Schools, in particular schools with poor drinking water access, may have been hesitant to participate in the study because of the observational component and the desire to remain inconspicuous. If this was the case, water access in FSAs may be lower than that reported in this study. In calculating the percentage of students who drank free water during mealtimes, we used student daily attendance as the denominator. In some schools, particularly high schools, students may have eaten meals off campus or in other areas of the schools, so we may have underestimated student consumption of free water at lunch. Finally, although in previous studies we examined students’ perspectives regarding access to drinking water in schools (14,18), we did not do so in this study.

Approximately half of schools had access to free drinking water in school FSAs before implementation of drinking water requirements, and in such schools, only 1 in 25 students drank the water. Increasing student water intake in schools requires a multipronged approach, which includes not only environmental changes (eg, installation of more appealing water delivery systems, such as hydration stations or dispensers), but also the promotion to encourage intake of water instead of SSBs. 
